# Irisin reduces the abnormal reproductive and metabolic phenotypes of PCOS by regulating the activity of brown adipose tissue in mice^†^

**DOI:** 10.1093/biolre/ioac125

**Published:** 2022-06-17

**Authors:** Yajing Zheng, Juan He, Dongyong Yang, Mengqin Yuan, Shiyi Liu, Fangfang Dai, Yifan Jia, Yanxiang Cheng

**Affiliations:** Department of Obstetrics and Gynecology, Renmin Hospital of Wuhan University, Wuhan, Hubei, China; Department of Obstetrics and Gynecology Ultrasound, Renmin Hospital of Wuhan University, Wuhan, Hubei, China; Department of Obstetrics and Gynecology, Renmin Hospital of Wuhan University, Wuhan, Hubei, China; Department of Obstetrics and Gynecology, Renmin Hospital of Wuhan University, Wuhan, Hubei, China; Department of Obstetrics and Gynecology, Renmin Hospital of Wuhan University, Wuhan, Hubei, China; Department of Obstetrics and Gynecology, Renmin Hospital of Wuhan University, Wuhan, Hubei, China; Department of Pain, Renmin Hospital of Wuhan University, Wuhan, Hubei, China; Department of Obstetrics and Gynecology, Renmin Hospital of Wuhan University, Wuhan, Hubei, China

**Keywords:** polycystic ovary syndrome, Irisin, brown adipose tissue, metabolism, uncoupling protein 1

## Abstract

Polycystic ovary syndrome (PCOS) is a common endocrine and metabolic disease in women, with clinical manifestations of anovulation and hyperandrogenaemia. The treatment of PCOS mainly focuses on improving clinical symptoms, such as insulin sensitivity or menstrual disorder, through drug treatment. However, due to the pathogenesis diversity of PCOS, there is still a lack of effective treatment in clinics. Metabolic disorder is the key factor in the occurrence of PCOS. Brown adipose tissue (BAT) is a special adipose tissue in the human body that can participate in metabolic balance by improving heat production. BAT has been demonstrated to be an important substance involved in the metabolic disorder of PCOS. Although increasing evidence indicates that BAT transplantation can improve the symptoms of PCOS, it is difficult to achieve BAT transplantation at present due to technical limitations. Stimulation of BAT activation by exogenous substances may be an effective alternative therapy for PCOS. In this study, we investigated the effects of Irisin on dehydroepiandrosterone (DHEA)-induced PCOS in mice and evaluated the effect of Irisin on serum hormone levels and changes in body temperature, body weight, and ovarian morphology. In our study, we found that Irisin can enhance the thermogenesis and insulin sensitivity of PCOS mice by activating the function of BAT. In addition, Irisin treatment can correct the menstrual cycle of PCOS mice, improve the serum steroid hormone disorder status, and reduce the formation of ovarian cystic follicles. In conclusion, our results showed that Irisin treatment significantly improved the metabolic disorder of PCOS and may provide a new and alternative therapy for the treatment of this pathology.

## Teaser

The mouse model proved that Irisin could improve the activity of BAT to treat PCOS.

## Introduction

Polycystic ovary syndrome (PCOS), an endocrine disease, is closely related to metabolic disorders and reproductive dysfunction. The incidence of PCOS ranges from 6 to 20%, affecting the physical and mental health of a wide range of women of reproductive age. There are currently no effective therapeutic measures [1]. PCOS clinical symptoms are highly heterogeneous and are mainly characterized by hyperandrogenism, ovarian polycystic changes, and chronic ovulatory disorders [2]. In addition, PCOS was also associated with obesity, insulin resistance (IR), type 2 diabetes mellitus (T2D), and cardiovascular disease (CVD) [3–7]. The etiology of PCOS is complex. Reproductive dysfunction and IR in PCOS patients are often closely related to the dysfunction of adipose tissue [8, 9]. Increasing evidence suggests that abnormal fat metabolism in PCOS patients is associated with IR, and improving the distribution of adipose tissue in the body can alleviate impaired ovarian function and systemic symptoms in PCOS mice [10–12]. Therefore, further exploration of the potential metabolic disorder mechanism of PCOS may provide new targets and a basis for the treatment of this syndrome.

Adipose tissue can be roughly divided into two categories. One is white adipose tissue (WAT), which is used for energy storage. Another is brown adipose tissue (BAT), which exists in humans and some other mammals [13]. BAT is a major site of nonshivering thermogenesis in mammals, which is low in content and activity in adults and can mediate thermogenesis by elevating the expression of uncoupling protein 1 (UCP1) [14] promoting systemic metabolic homeostasis through cytokines such as adiponectin and fibroblast growth factor 21 (FGF21) [15]. Thermogenesis through adipose tissue is an important link in energy metabolism that can improve the occurrence and development of a variety of metabolic diseases by regulating mitochondrial function [16]. In addition, studies have reported that BAT levels are reduced in PCOS patients and that BAT thermogenesis is inversely correlated with androgen levels [17, 18]. The increase and activation of BAT may serve as a promising strategy for the treatment of metabolic diseases such as PCOS. Animal experiments demonstrated that activation of BAT improved PCOS symptoms in rats [19]. Furthermore, transplantation of exogenous BAT reverses IR, obesity, and reproductive dysfunction by upregulating adiponectin expression and enhancing energy expenditure in PCOS animal models [20, 21]. However, with respect to current medical conditions, BAT transplantation is not achievable for clinical applications. Hence, activating the expansion and activity of endogenous BAT is an alternative strategy for PCOS therapy. Endogenous BAT can be converted from abundant WAT, a process known as “browning” [16, 22]. Therefore, stimulating browning may be a new research topic in the treatment of PCOS.

Irisin, a thermogenic adipose agonist protein and polypeptide hormone, is cleaved by fibronectin type III domain containing protein 5 (FNDC5) and subsequently released into the extracellular milieu to exert its effects. It is expressed in a variety of cells and tissues, including skeletal muscle, adipocytes, and the heart [23]. However, the latest study found that Irisin is also produced in the ovaries and endometrium [24]. Irisin regulates the expression of energy expenditure markers such as mitochondrial UCP1 and peroxisome proliferator activated receptor gamma coactivator 1 alpha (PGC-1 alpha) by binding to a variety of cell surface receptors (integrin receptor family) [25] and participates in various physiological and pathological processes, such as BAT activation [23, 26]. Irisin can significantly elevate FGF21 (a key adipocyte browning factor) expression [26], enhance BAT activation, and induce BAT production, contributing to the amelioration of metabolic disorders in obese mice [27]. However, there is no information about the role of Irisin in PCOS. Therefore, we hypothesize that Irisin may improve the metabolic and reproductive dysfunction of PCOS by activating BAT.

Our study used a dehydroepiandrosterone (DHEA)-induced PCOS mouse model as a research object to explore the function of Irisin in PCOS mice, the relationship between Irisin and metabolic disorders, and the possible pathological mechanism involved in regulating PCOS. The objective of this study was to verify the therapeutic effect of Irisin and explore its mechanism. Our investigation may provide new research ideas and therapeutic targets for the diagnosis and treatment of PCOS.

## Materials and methods

### Animals

C57BL/6 J (3-week-old) female mice, weighing 15–20 g, were purchased from Beijing Vitalriver Laboratory Animal Technology Co Ltd., China. Experimental animals were randomly divided into control (glycerol treatment) and experimental groups (PCOS, DHEA treatment). Five mice per cage were housed in the animal experimental center of Renmin Hospital of Wuhan University under constant environmental conditions with a 12-h light–dark cycle and with an unscheduled supply of water and food. All animal studies were approved by the Ethics Committee for Laboratory Animal Welfare (IACUC) of Renmin Hospital of Wuhan University (No. WDRM animal (f) No. 20200908).

### Induction of PCOS

Dehydroepiandrosterone (DHEA) was used to induce PCOS in mice as previously mentioned [28]. DHEA used for establishing the PCOS model was purchased from Yangzhou Pharmaceutical Co Ltd., China (cat. No. h10940064). Female mice (3 weeks old) were subcutaneously injected with DHEA (60 mg/kg, *n* = 40) dissolved in 0.1 mL glycerol. The control group (3 weeks old) was subcutaneously injected with 0.1 mL glycerol (*n* = 20). The above animals were injected daily for 3 weeks. Successful PCOS model mice were selected based on the evaluation of the estrous cycle by 12 consecutive days of vaginal cytology, as well as the analysis of serum hormones and ovarian morphology.

Mice in each group underwent OGTT and ITT on the morning of the second and third days after all treatments. The mice were sacrificed, and samples were collected. Serum was collected at diestrus for sex hormone and Irisin determination.

### Irisin treatment

All mice were treated after successful induction of the PCOS model. Female mice in Irisin-treated groups (DHEA + Irisin, *n* = 10) were treated with Irisin [29] (500 μg/kg, Irisin, human recombinant, CAS 7263-10, Biovision) by intraperitoneal injection once/day for 14 consecutive days. The placebo group (DHEA + NaCl, *n* = 10) was injected with saline solution (0.1 mL) once/day for 14 consecutive days. The inhibitor group (DHEA + Irisin + CWHM-12, *n* = 10) used the integrin alpha V (ITGAV) inhibitor CWHM-12 (5 μg/kg, m00526; Biomart) to antagonize the effects of Irisin, and CWHM-12 was administered once/day for 14 consecutive days. The mice in the control group (control group, *n* = 10) were from glycerol-treated mice, and normal saline (0.1 mL) was administered once a day for 14 days. Subcutaneous injection was used for the generation of mouse models, while intraperitoneal injection was used for intervention treatment.

At the end of the treatment, six mice were randomly selected from each group, and one ovary of each mouse was stained with H&E. The subcutaneous adipose tissue of each group and the remaining ovary was rapidly frozen and stored at −80°C for subsequent studies.

### Assessment of the estrous cycle

After DHEA injection, vaginal smears were taken every day at 09:00 from the 10th to the 21st day. After Irisin treatment was administered, vaginal smears were taken daily at 09:00 from day 3 to day 14. The stages of the estrous cycle were determined by microscopic analysis of the major cell types in vaginal smears stained with H&E according to previously validated methods [30, 31].

### Oral glucose tolerance test and insulin tolerance test

Female mice were fasted for 12 h (21:00-09:00) before the OGTT experiment and 4 h (09:00-13:00) before the ITT experiment with free access to water. Blood glucose levels were determined from the tail vein using an Accu-Chek Performa (Roche Diagnostics) glucometer. After measurement of fasting blood glucose, D-glucose (2.0 g/kg) or insulin (1 U/kg) was administered, and tail vein blood was taken at 15, 30, 60, 90, and 120 min after glucose or insulin administration to measure blood glucose levels. The total area under the curve (AUC) of the glucose response was calculated using GraphPad Prism 7.0 software.

### Detection of body temperature in mice

The mice (2 per cage) were placed in a cold room (4°C) for up to 4 h with free access to food and water. A veterinary electronic thermometer (Zhengzhou Haorunqi Electronic Science and Technology Co Ltd.) was used to measure rectal temperature in mice. Rectal temperature was measured after 4 hours of cold stimulation. The experiment is generally started at 7 a.m. and measured at 11 a.m. Each mouse was repeated three times to ensure the accuracy of the experimental results. At ambient temperature, the rectal temperature of the mice was measured at 11 a.m. the next day. Similarly, each mouse was measured three times.

### Micro PET-CT

PET-CT imaging was performed using a small animal PET system (Ruipai Ning Technology Co Ltd, Suzhou, China) at the PET center of Union Hospital Tongji Medical College Huazhong University of Science and Technology. Female mice were fasted overnight with free access to water, lightly anesthetized with 2% isoflurane, and through the tail vein 18F-FDG was administered (200 μCi). Female mice were scanned starting 60 min after drug injection with PET first and CT second. PET was in static mode, with a whole-body scan at 10 min; CT showed a normal pattern. PET static scans were acquired for 10 min, and images were reconstructed using the osem3d algorithm. PET images were analyzed with Inveon Research Workplace (IRW) software to outline three-dimensional regions of interest (ROIs) to measure tracer uptake rates. The number of individuals with 18F-FDG uptake per ROI was calculated. The cumulative data of 18F-FDG on microscopic PET images were expressed as the standardized uptake value (SUV), which was determined by dividing the relevant ROI concentration by the ratio of injected activity to body weight, using the formula SUV = (CT/DInj) × (VT/WT) × WS (where CT is the activity within a unit volume of tissue, DInj is the injected dose, VT is the volume of tissue, and WT is the mass of tissue). Here, the density of tissue VT/WT was set to 1 and WS is the mass of mice.

### Blood analysis

Blood samples were collected by cardiac puncture under anesthesia. Blood samples were centrifuged at 3000 rpm, and serum was separated and stored at −80°C for subsequent blood analysis. ELISA kits were used to measure mouse plasma follicle stimulating hormone (FSH) (CSB-E12770 m, CUSABIO), luteinizing hormone (LH) (CSB-E06871 m, CUSABIO), testosterone (T) (CSB-E05101 m, CUSABIO), anti-Müllerian hormone (AMH) (CSB-E13156 m, CUSABIO), and Irisin (DRE-M9930c, Kmaels) levels.

### H&E staining and immunohistochemistry

The ovaries of each group (*n* = 6) were treated by the same way. Ovarian tissues were quickly collected, fixed in 4% paraformaldehyde overnight at room temperature and stored in fresh 75% ethanol at 4°C. Ovaries were dehydrated and paraffin embedded. The sections were prepared and stained with H&E. The ovaries were longitudinally and serially sectioned into 5-μm sections. All of the sections were mounted onto a glass slide and observed for histomorphological analysis under a light microscope (Nikon, Tokyo, Japan). The number of corpora lutea and cystic follicles was counted according to ovarian morphology.

### Western blotting

Adipose tissues were disrupted in RIPA buffer (1.0% Triton X-100, 150 mM sodium chloride, 0.5% sodium deoxycholate, 0.1% SDS, 50 mM Tris, protease, and phosphatase inhibitor mixture (Roche Diagnostics)). The tissues were washed twice with cold PBS and collected in 100 μL of 1x hypotonic buffer containing 20 mM Tris–HCl of pH 7.4, 10 mM NaCl, and 3 mM MgCl2 followed by incubation on ice for 15 min. Five microliter detergent (10% NP40) was added and vortexed for 10 s, then centrifuged for 10 min at 3000 rpm at 4°C. The supernatant contained the cytoplasmic fraction. The remained nuclear pellets were re-suspended in 50 μL cell extraction buffer presupplemented with 1 mM PMSF and protease inhibitor cocktail and ultrasonicated, followed by centrifugation for 30 min at 14,000 g, 4°C. Supernatant was collected as the nuclear fraction. Protein concentrations were determined by a BCA assay kit (Pierce Diagnostics, Servicebio, Wuhan, China). Protein was separated by 10% (w/v) SDS/PAGE, transferred to a PVDF membrane (Millipore), blocked in 5% (w/v) skim milk (OXOID) in TBST (0.02 M Tris base, 0.1% Tween 20, 0.14 M NaCl pH 7.4), incubated with primary antibodies overnight at 4°C and then incubated with secondary antibodies conjugated with HRP. The primary antibodies used were: anti-UCP1 (1:1000, ProteinTech), anti-ITGAV (1:1000, ABclonal), and anti-tubulin (1:1000, Abmart). Signals were detected with SuperSignal West Pico chemiluminescent substrate (Pierce). ImageJ software was used for quantitative analysis of WB bands.

### Statistical analysis

GraphPad Prism version 7.0 (GraphPad Software) and SPSS version 23.0 were used for statistical analysis. No data were excluded from the data analysis. To test normality, the Shapiro–Wilk test was performed, and then, depending on its outcome, one-way ANOVA with Tukey’s post hoc test was used to evaluate the statistical significance of differences among three or more groups. A two-tailed Student’s *t*-test was used to evaluate statistical significance between two groups in glucose dynamics. Data are shown as the means ± SEM. *P* < 0.05 was considered a statistically significant difference.

## Results

### Establishment of the DHEA-induced PCOS mouse model

To demonstrate the effect of Irisin on PCOS, we subjected mice to subcutaneous injection of DHEA for 21 consecutive days, thus obtaining the PCOS model for subsequent studies (Supplementary Figure S1a). To evaluate whether the mouse model of PCOS was established successfully, we examined the body weight, estrous cycle, ovarian morphology, and serum hormone levels of mice in the PCOS group (DHEA) and control group mice. The mice were weighed daily during the modelling period, and it was found that by the end of the experimental protocol, the body weight of mice in the PCOS (DHEA) group was significantly higher than that in the control group (Supplementary Figure S2a and b). We used the vaginal smear method to perform smear microscopy of vaginal secretions in mice for 12 consecutive days and analyzed the vaginal cytology of mice. Representative images of vaginal cytology at each estrous cycle stage are shown in Figure 3b. Additionally, we analyzed the proportion of mice in each stage of the estrous cycle and found that the control group mice had 4 continuously changing stages of the estrous cycle (Supplementary Figure S2c). The whole estrous cycle lasted approximately 4-5 days, and the proportion of mice in each stage was balanced (Supplementary Figure S2d). However, the estrous cycle of mice in the PCOS group showed irregular changes (Supplementary Figure S2c), and the estrous interval of PCOS mice was prolonged or in a stagnant state (Supplementary Figure S2e). The core features of PCOS are polycystic ovaries and chronic ovulation disorder, and we observed alterations in mouse ovarian tissue morphology with a reduced number of corpora lutea and the appearance of cystic dilated follicles (Supplementary Figure S2f) in the ovaries of PCOS mice. However, no cystic follicles were found in the ovaries of control mice, and the morphology of follicles was regular (Supplementary Figure S2f). Compared with the control group, the number of corpora lutea in the PCOS group decreased significantly (Supplementary Figure S2g), while the number of cystic follicles increased significantly (Supplementary Figure S2h). The serum levels of hormones such as T, AMH, LH, and FSH were examined, and LH/FSH was calculated. Serum levels of T, AMH, and LH were greatly higher and LH/FSH ratio was >3 (Supplementary Figure S2i-m) in PCOS mice compared to controls. There was no significant difference in serum FSH between the two groups (Supplementary Figure S2l). These results indicated that we successfully constructed a PCOS mouse model, and we screened a cohort of mice for subsequent studies.

### The Irisin serum levels in PCOS mice were decreased

**Figure 1:**
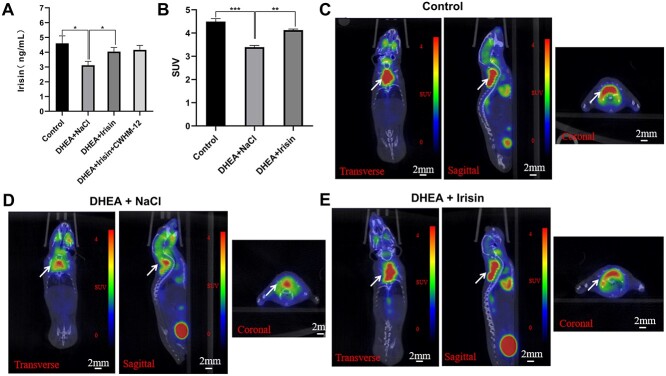
Irisin treatment can increase Irisin content and BAT activity in DHEA induced PCOS mice. (A) Quantitative analysis of Irisin level in the serum of mice (n = 8 mice per group). (B) Standard uptake value (SUV) of PET-CT (n = 3 mice per group). (C, D, E) PET-CT was used to detect activated BAT in mice, (C) PET-CT representative pictures of Control group, (D) PET-CT representative pictures of DHEA + NaCl group and (E) PET-CT representative pictures of DHEA + Irisin group. From left to right are horizontal plane, sagittal plane and coronal plane. The position indicated by the white arrow is BAT. Data were analyzed using one-way ANOVA with Tukey’s multiple comparison post-hoc test and data are presented as means ± SEM. ^*^P < 0.05; ^**^P < 0.01; ^***^P < 0.001.

There is currently no clear information about the alteration of Irisin content in PCOS, so to better investigate whether Irisin treatment has benefits in DHEA-treated PCOS mice, we examined the change in Irisin serum levels in PCOS mice (Supplementary Figure S1b). Serum from 10 control and 10 PCOS mice was collected for analysis. We found that serum Irisin of PCOS mice was significantly lower than that in the control group, and Irisin treatment significantly increased plasma levels of Irisin in PCOS mice. After Irisin treatment, the concentration of Irisin in PCOS mice was close to that in the control group, and this effect could not be inhibited by ITGAV antagonists (Figure 1a). This proves our conjecture that there is an association between the pathogenesis of PCOS and Irisin plasma concentration. Therefore, we conducted follow-up experiments to verify the effects of restore plasma levels of Irisin on PCOS mice. After exogenous treatment with Irisin, we observed the signs on mice. In our study, integrin receptor inhibitors did not affect the content of Irisin.

### Irisin enhances BAT activity in polycystic ovary syndrome mice

More evidence suggests that PCOS is closely related to BAT activity. IR is often accompanied by a reduction in BAT activity [17], and enhancement of BAT activity may play a critical role in the treatment of PCOS [20]. In previous studies of Irisin, it has been demonstrated that Irisin has an effect on BAT, and Irisin also has a certain effect in metabolic diseases. In order to verify the correlation between the decrease of BAT activity in PCOS and the decrease of Irisin, and whether the metabolic disorder of PCOS is related to the decrease of Irisin, we conducted follow-up experiments [27, 32]. Therefore, we hypothesized that alteration of plasma Irisin could reverse the phenotype of PCOS by elevating the activity of BAT. Although the adipose tissue in adult mice is basically white, many studies have shown that WAT can brown into BAT under certain conditions and improve the activity of BAT. Cold, thyroid hormones and some drugs have been proven to play important roles in the browning of WAT. The activity of BAT can be indirectly detected by PET-CT and monitoring body temperature under cold conditions [33, 34].

We subject PCOS mice to Irisin treatment for two weeks (DHEA + Irisin) to observe the effects of Irisin on this model (Supplementary Figure S1b). It has been shown that Irisin regulates the activity of BAT mainly through ITGAV [25], and CWHM-12 is a specific ITGAV antagonist [35]. BAT activity was detected with positron emission tomography computed tomography (PET-CT) (Figure 1b). The results showed that the BAT activity of mice in the PCOS (DHEA + NaCl) group was significantly lower than that in the control group (Figure 1c and d). Irisin treatment significantly increased endogenous BAT activity (Figure 1d and e) to the level of the control group. These results indicate that the role of Irisin in PCOS mice is closely related to the activity of BAT.

**Figure 2:**
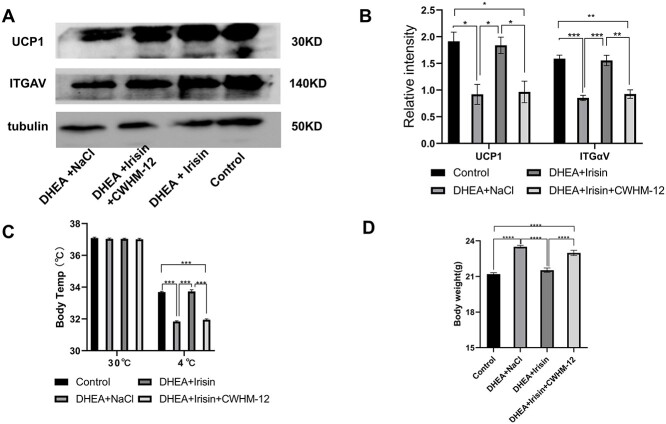
Irisin treatment increased BAT related protein and thermogenic capacity, and decreased the body weight of DHEA induced PCOS mice. For A-D, the mice were divided into four groups (control, DHEA + NaCl, DHEA + Irisin, DHEA + Irisin + CWHM-12). (A) Representative Western blots of UCP1 and ITGAV in adipocytes of mice in each group. (B) Quantitative analysis of UCP1 and ITGAV. The gray value of immunoblotting protein band in figure A was analyzed by Image J software. (C) Body temperature changes of mice in normal environment and cold exposure (*n* = 10 mice per group). (D) Body weight changes of four groups of mice (*n* = 10 mice per group). Data were analyzed using one-way ANOVA with Tukey’s multiple comparison post hoc test and data are presented as means ± SEM. ^*^P < 0.05; ^**^P < 0.01; ^***^P < 0.001.

The body temperature and BAT specific molecules of mice were analyzed to identify the effects of Irisin on BAT activity in PCOS mice. We analyzed the expression levels of the UCP1, a BAT-specific gene, to assess the thermogenic activity of BAT. UCP1 is only expressed in BAT, not in WAT, and an increase of UCP1 expression can reflect the activity of BAT [15]. The results showed that UCP1 expression in the DHEA + Irisin group was sharply higher than that in the DHEA + NaCl group (Figure 2a and b), and the expression of UCP1 was close to that of the control group, which was consistent with the PET-CT results. The effect of Irisin on UCP1 expression was antagonized by CWHM-12. After CWHM-12 treatment, the expression of UCP1 decreased significantly compared with the control group, which was the opposite effect of Irisin treatment. In addition, Irisin treatment significantly increased the content of ITGAV in DHEA induced PCOS mice, which was significantly inhibited by CWHM-12. These results suggest that the effect of alteration of plasma Irisin concentration on BAT activity may be mediated through integrin alpha V receptor pathway.

BAT plays a role by increasing heat production. Its activity often does not change in normal environment, but BAT can be activated under cold conditions. In the detection of BAT activity, a change of body temperature can indirectly reflect the activity of this tissue by exposing to cold environment [33]. In our study, there was no difference in body temperature of mice in different groups under normal feeding environment. However, when mice in each group were exposed to 4°C environment for 4 h, and the body temperature was measured, we found that Irisin treatment significantly increased the body temperature in PCOS mice (Figure 2c), and this effect could be antagonized by CWHM-12. Under cold exposure, the body temperature of mice treated with Irisin was close to that of the control group, while the body temperature of mice treated with CWHM-12 was lower than that of the control group. These results suggest that Irisin treatment elevates BAT activity and increases thermogenesis. The role of increased of plasma Irisin concentration in PCOS is related to the activity of BAT, which is mediated by the ITGAV pathway.

The roles of alteration of plasma Irisin concentration in the reproductive and metabolic phenotypes of PCOS mice were studied. The body weights of the mice in each group were compared. Irisin treatment reduced the body weight of PCOS mice, and this effect was reversed by CWHM-12. Compared with the control group, Irisin treatment effectively restored DHEA-induced weight gain to normal. CWHM-12 treatment did not reduce DHEA-induced body weight (Figure 2d). These results effectively show that Irisin restored body weight of POCS through ITGAV.

### Irisin treatment reverses estrous acyclicity and endocrine disturbances in PCOS

**Figure 3:**
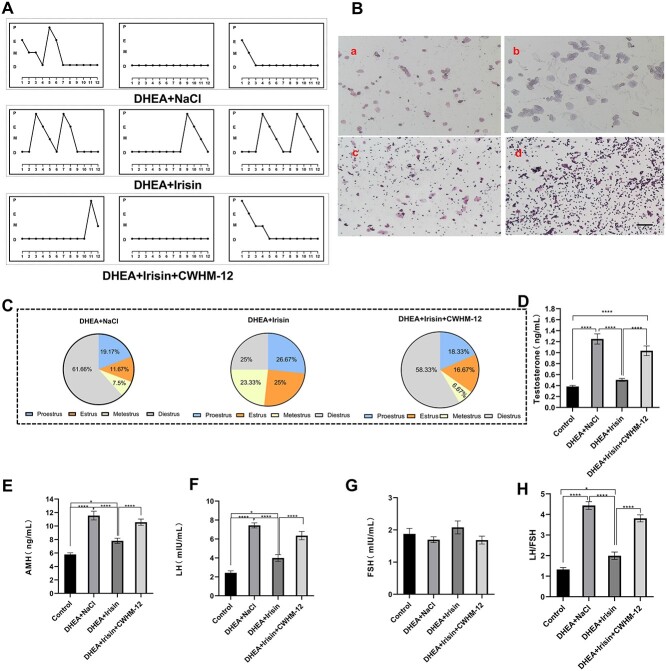
Irisin treatment improved the estrous cycle and hormone level disorder of PCOS mice induced by DHEA. For A-H, the mice were divided into four groups (control, DHEA + NaCl, DHEA + Irisin, DHEA + Irisin + CWHM-12). For A-C: Irisin treatment significantly improved the estrous cycle disorder of PCOS mice induced by DHEA. (A) The estrous cycle of mice (*n* = 10 mice per group) was observed for 12 days. P: proestrus; E: estrus; M: metestrus; D: diestrus. (B) H&E stained vaginal secretion smears at four stages of mouse estrous cycle (magnification: 100x). a: proestrus; b: estrus; c: metestrus; d: diestrus. (C) Proportion of estrous cycle stages in mice treated with Irisin or CWHM-12. (D) Testosterone level (*n* = 8 mice per group). (E) Anti Mullerian hormone (AMH) level (*n* = 8 mice per group). (F) Luteinizing hormone (LH) level (*n* = 8 mice per group). (G) Follicle stimulating hormone (FSH) level (*n* = 8 mice per group). (H) LH/FSH ratio. Data were analyzed using one-way ANOVA with Tukey’s multiple comparison post-hoc test and data are presented as means ± SEM. ^*^P < 0.05.

Studies have shown that PCOS mice ovulate irregularly and exhibit alterations in estrous cycles. We investigated whether Irisin treatment could relieve the irregular estrous cycle in PCOS mice. The estrous cycle is mainly determined by continuous vaginal smears. Figure 3b is a representative image of each stage of the estrous cycle. According to the analysis of cell morphology, the normal estrous cycle has four consecutive stages, and one estrous cycle lasts for 4-5 days. The estrous cycle disorder of PCOS is mainly characterized by remaining in a stage without continuous changes, and the change of each stage is irregular. The estrous cycle in the control group was normal and evenly distributed in each stage (Supplementary Figure S2c and d). The estrous cycle of DHEA + NaCl group mice was irregular and maintained at a fixed stage for a long time, and the distribution of each stage was uneven. Irisin treatment significantly improved DHEA-induced estrous cycle disorder, which was blocked by CWHM-12. After CWHM-12 treatment, the estrous cycle of mice was irregular, and the distribution of each stage was uneven (Figure 3a, c and Supplementary Table S1). Thus, increased of plasma Irisin concentration can improve the estrous cycle disorder of PCOS through ITGAV.

In the study of PCOS, the therapeutic effect of drugs on PCOS is often observed through changes in hormone levels. The serum hormone levels of mice in each group were determined to evaluate the effect of Irisin treatment on the PCOS mouse model. PCOS patients showed hyperandrogenaemia, increased AMH levels, and increased LH levels, but FSH levels showed no change. In addition, PCOS was determined by detecting the ratio of LH/FSH [36, 37]. The results showed that the T, AMH and LH levels in PCOS mice decreased after Irisin treatment, but the FSH level did not change significantly (Figure 3d-h and Supplementary Table S2). The LH/FSH ratio > 3 in DHEA- induced PCOS mice, decreased to less than 2 after Irisin treatment. After Irisin treatment, the hormone levels were similar to those in the control group. However, the levels of AMH and LH were still higher than those in the control group. This may be because Irisin treatment did not completely improve the hormone disorder of PCOS, which may be related to the dose and action time of Irisin. Compared with the control group and Irisin treatment group, the hormone levels of the CWHM-12 group were greatly different, and the FSH level was excluded here. Taken together, these results indicate that increased of plasma Irisin concentration treatment reverses the irregular estrous cycle and endocrine disorder in PCOS mice and that this effect is exerted through ITGAV.

### Irisin treatment improves ovarian function in PCOS

PCOS mice often exhibit ovarian morphological changes. To observe the effect of Irisin on ovarian morphology and function, we collected the ovaries of mice in each group for ovarian morphology analysis.

**Figure 4:**
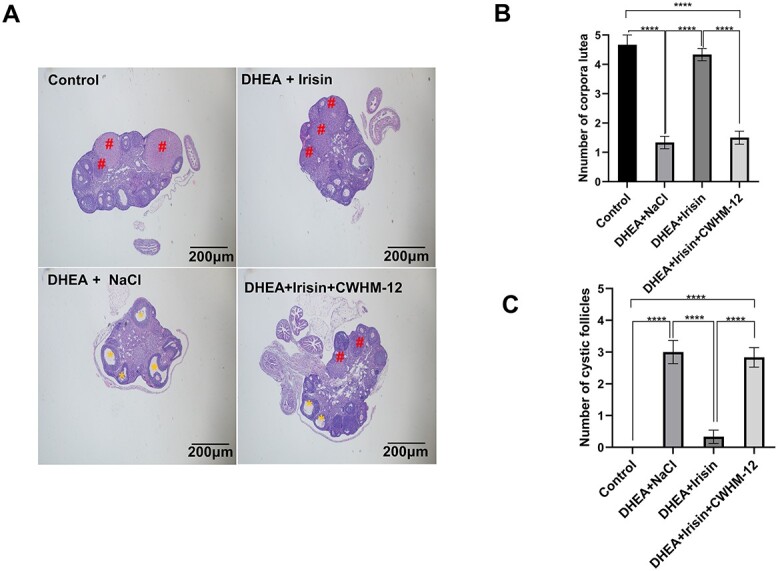
Irisin improved ovarian dysfunction in DHEA induced PCOS mice. For A-F, the mice were divided into four groups (control, DHEA + NaCl, DHEA + Irisin, DHEA + Irisin + CWHM-12). (A) H&E staining of representative ovaries (*n* = 6 mice per group). Scale bar: 200 μm (magnification: 40×). Yellow asterisk marks represent cystic follicles and red well marks represent corpus luteum (CLs). (B) Quantitative analysis of corpora lutea. (D) Quantitative analysis of cystic follicles. Data were analyzed using one-way ANOVA with Tukey’s multiple comparison post-hoc test and data are presented as means ± SEM. ^*^P < 0.05; ^**^P < 0.01.

In the control group, the ovarian histology was normal, characterized by a follicular morphology and corpora lutea number regular. However, DHEA-induced PCOS mice showed cystic expansion of follicles and a decreased number of corpora lutea. Compared with the DHEA + NaCl group, the number of corpora lutea increased and the number of cystic follicles decreased in Irisin-treated mice, being close to those in the control group. In contrast to Irisin, the number of corpora lutea in mice treated with CWHM-12 was significantly lower than in the control group, while the number of cystic follicles increased, which was similar to that in the DHEA + NaCl group (Figure 4a-c and Supplementary Table S3). By observing whether there are cystic follicles in ovarian sections and the changes in the number of corpora lutea, we can indirectly judge the ovulation function. PCOS patients have polycystic ovarian morphology [38], which we have also observed in this animal model.

These results showed that increased of plasma Irisin concentration reversed ovarian polycystic changes and improved ovarian function in PCOS and that its effect was exerted through ITGAV.

### Irisin reverses metabolic abnormalities in PCOS

In previous experiments, we demonstrated that Irisin can improve ovary morphology and body weight in PCOS mice DHEA induced by increasing the activity of BAT though integrin signaling pathway. Next, we studied the effect of Irisin on insulin sensitivity in PCOS. PCOS is characterized by IR. To assess insulin sensitivity, we performed an OGTT and ITT to evaluate whether Irisin can affect glucose tolerance in PCOS mice.

**Figure 5:**
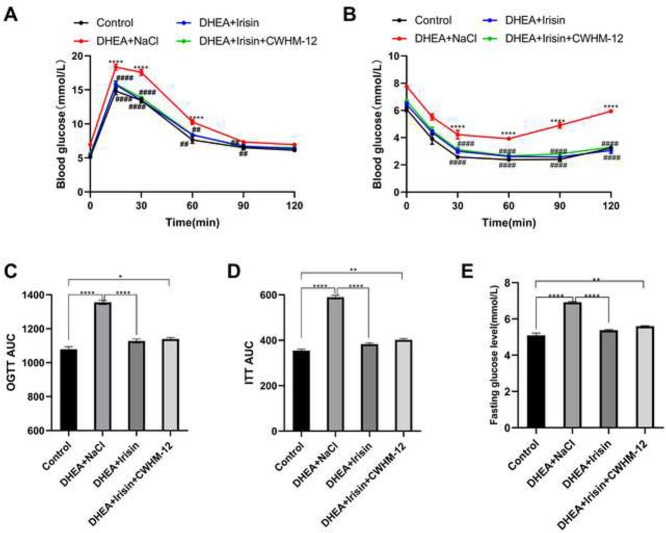
Irisin improved insulin sensitivity in DHEA induced PCOS mice. For A-E, the mice were divided into four groups (control, DHEA + NaCl, DHEA + Irisin, DHEA + Irisin + CWHM-12). (A) OGTT (*n* = 5 mice per group). (B) ITT (*n* = 5 mice per group). (C) Area under curve (AUC) of OGTT. (D) Area under curve (AUC) of ITT. (E) Quantitative analysis of fasting blood glucose (*n* = 5 mice per group). Data were analyzed using one-way ANOVA with Tukey’s multiple comparison post-hoc test, the glucose dynamics between the groups, the test that should be used is a two-way ANOVA and data are presented as means ± SEM. ^*^P < 0.05; ^**^P < 0.01 versus the control. ^#^*P* < 0.05; ^##^*P* < 0.01 versus the DHEA + NaCl.

OGTT and ITT were performed in mice, and the blood glucose curve was drawn. The results showed that the blood glucose levels of PCOS mice at 15 min and 30 min after oral glucose administration were significantly higher than those of the control group, whereas the blood glucose levels of Irisin treated group was lower than the PCOS group and close to that of the control group (Figure 5a). The glucose AUC during OGTT in the PCOS group was significantly higher than the control group (Figure 5c), and the glucose AUC of Irisin treated group decreased compared with the PCOS group, indicating that Irisin can improve the impaired glucose tolerance in PCOS mice. The blood glucose levels were also greatly changed after insulin administration. We found that the blood glucose levels were higher in the PCOS group compared with the control group, and it was lower in the Irisin treated group (Figure 5b). The glucose AUC during ITT was higher in the PCOS group than in the control group, whereas it was significantly lower in the Irisin group (Figure 5d). In addition, those results show that fasting blood glucose levels were significantly higher in the PCOS group compared with the control group, whereas the Irisin treatment group showed lower fasting blood glucose levels (Figure 5e). Surprisingly, CWHM-12 did not significantly affect the effect of Irisin on blood glucose. Compared with the control group, there was a significant difference in blood glucose levels after CWHM-12 treatment, but there was no significant difference between the Irisin treatment group and the control group. This indicates that the antagonistic effect of CWHM-12 on Irisin plays a weak role in the regulation of blood glucose. We speculate that the reason for this result may be because CWHM-12 demonstrates high potency against the possible alpha and beta subunit binding partners (AVB1, AVB3, AVB6, and AVB8) and is slightly less potent against AVB5 than against other AVB integrins. In the regulation of blood glucose, AVB5 plays a greater role than other AVB integrins. These results suggested that Irisin effectively improved insulin sensitivity and abnormal glucose tolerance in mice with PCOS induced by DHEA.

## Discussion

PCOS patients are often accompanied by obesity, IR and other metabolic abnormalities. Previous reports has shown that adipose tissue plays an important role in the treatment of PCOS, and it is beneficial for improving body weight or metabolic disorders in PCOS patients [1, 8, 39]. BAT, as a specialized adipocyte, plays a thermogenic function in the body and has a key role in energy metabolism [40]. BAT activity effectively ameliorated IR and obesity, reversed metabolic disorders, and reduced body weight in obese mice [41, 42]. Studies have shown that BAT activity is decreased in PCOS patients, and the reduction in energy expenditure in PCOS patients may be related to a decline in BAT function. As shown in the current study, the BAT activity of PCOS mice decreased significantly. At present, research about PCOS and BAT mainly focuses on three aspects: the activity and role of BAT in PCOS, exogenous implantation of BAT to study the relationship between PCOS and BAT, and activation of BAT by cold or electrical stimulation. However, there are few relevant studies, and further discussion is needed. BAT transplantation currently has certain risks and is difficult to achieve clinically, and seeking alternative methods to enhance the activity of endogenous BAT has become an ideal way to treat PCOS. Therefore, we investigated whether Irisin could ameliorate the symptoms of PCOS by activating endogenous BAT.

**Figure 6:**
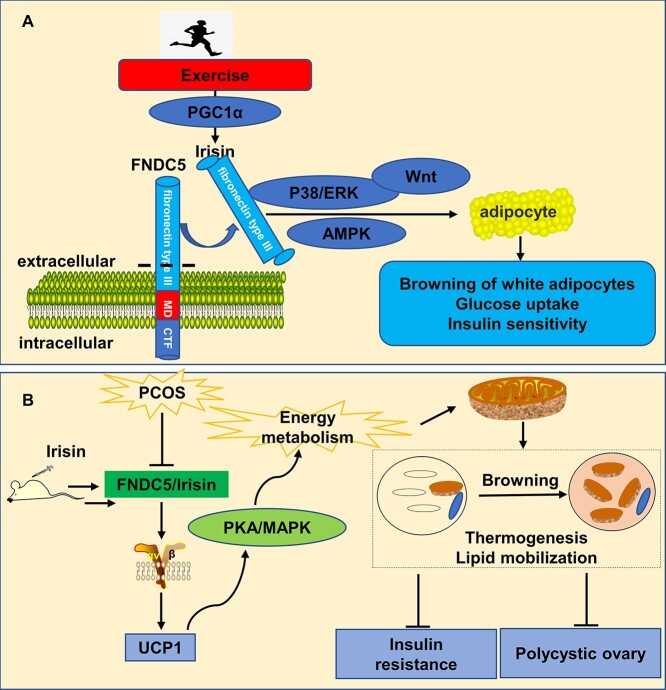
Schematic of hypotheses concerning the mechanism underlying of Irisin-BAT in regulating DHEA-induced PCOS mice. (A) Production and main function of Irisin. (B) Mechanism of Irisin in DHEA induced PCOS mice.

Irisin, a novel adipokine, is a thermogenic protein produced by FNDC5 cleavage. It increases thermogenesis and promotes energy consumption by browning WAT to BAT [43, 44]. In this study, we evaluated the effect of Irisin on BAT (Figure 6a). By observing the induction of BAT by Irisin, the change in body temperature, and the expression of the BAT-specific protein UCP1, we found that Irisin can effectively improve the activity of BAT and it is consistent with previous studies. Most of the previous studies on Irisin focused on exercise and the cardiovascular and nervous systems. Irisin can increase the survival of bone cells and the production of sclerotin, improve myocardial injury, and participate in the production of the central nervous system [25, 45, 46]. With the deepening of research, there are an increasing number of studies on Irisin in metabolism-related diseases, and we focused on the relationship between Irisin, BAT and PCOS. The absence of Irisin can inhibit the browning of adipose tissue and impair insulin sensitivity [47]. Combined with the fact that PCOS patients often have IR and decreased BAT activity, we speculate that Irisin can play a role in the treatment of PCOS. The results showed that the level of Irisin in PCOS mice induced by DHEA decreased, which indicated that the reduction of Irisin could be related to the pathogenesis of PCOS. Our results are consistent with Wang et al., where the level of serum Irisin in PCOS patients was significantly decreased [48]. FNDC5 is often knocked out in relevant studies to obtain a mouse model of Irisin deficiency. When Irisin is absent, the mortality of mice increases, and fertility decreases [49], which indicates that Irisin may play a vital role in PCOS. Our results confirm that restoring plasma irisin concentration to normal levels can improve body weight and thermogenic capacity of PCOS and promoted fat metabolism by increasing the activity of BAT (Figure 6b).

In this study, we used a DHEA-induced PCOS mouse model for the experiment. DHEA, the major androgen precursor, can be transformed into androstenedione (A4) and T by biological enzymes *in vivo* and does not directly cause elevation of androgen. The DHEA-induced PCOS mouse model is currently one of the most widely used animal models owing to its high stability [50, 51]. The administration of exogenous Irisin effectively increased the content of Irisin in DHEA-induced PCOS mice, making up for the decrease by Irisin itself. After Irisin treatment, the weights of PCOS mice decreased, and BAT activity increased. Irisin may increase energy consumption through a large amount of fat mobilization, which is reflected in the increase in body temperature. Insulin sensitivity is closely related to the occurrence and development of PCOS. We carried out OGTT and ITT experiments, and the results showed that Irisin treatment highly improved IR in DHEA-treated PCOS mice. This result is consistent with the previously mentioned reduction in insulin sensitivity by Irisin deficiency.

A significant feature of PCOS patients is hyperandrogenemia. In addition, the serum AMH and LH levels of PCOS increased significantly. The LH/FSH ratio was also calculated in this study. When LH/FSH ratio > 2, clinicians should be alert to the occurrence of PCOS. The change in PCOS is not only a disorder of hormone levels but also a change in ovarian morphology [52]. The ovary of POCS shows cystic changes, which can be observed by ovarian section or imaging. Due to the limitation of technology, morphological changes in the ovary were observed only by ovarian section staining in this work. Irisin treatment significantly improved the hormone disorder and ovary morphology in DHEA-induced PCOS mice. Studies on PCOS mice showed that Irisin played an important role in the treatment of PCOS and significantly improved the reproductive-related phenotype of PCOS mice induced by DHEA.

We studied the role of the integrin receptor in the effect of Irisin on PCOS. By using CWHM-12, an integrin receptor inhibitor, we found that the effect of Irisin was inhibited after blocking the integrin signaling pathway. Therefore, we speculate that the effect of Irisin on PCOS is mediated by the integrin signaling pathway after promoting the activity of BAT. However, in the experiments about glucose metabolism, the antagonistic effect of CWHM-12 on Irisin action was not observed. This may be due to the therapeutic dose and timing of Irisin or because CWHM-12 does not completely block the effect of integrin signaling pathway.

Although our experiment has achieved some results, due to technical limitations, we only verified the effect of Irisin using a DHEA-induced PCOS animal model. In addition, this model has been widely recognized in simulating the symptoms of PCOS, but it is still different from real patients, so we cannot fully prove that Irisin may play a role in PCOS patients. In the future, we will conduct deeper research on fertility and other aspects of this syndrome. At the same time, we will continue to explore the specific action mechanism of Irisin, not just ITGAV. In view of the important roles of the P38 and ERK1/2 signaling pathways in Irisin-mediated fat metabolism [53], we will further discover their relationship with ITGAV and explore the specific action mechanism of Irisin in PCOS mice and even patients.

## Conclusion

We demonstrate for the first time that Irisin imbalance could be associated with endocrine and metabolic disorders in PCOS mice induced by DHEA. Irisin treatment improves insulin sensitivity, disorders in steroid hormone secretion, ovarian morphology, and function in PCOS mice induced by DHEA. Irisin elevates UCP1 expression in the ovary and improves the metabolic and reproductive features of PCOS mice by increasing the activation of BAT. The present study provides new insights into the potential role of Irisin in PCOS treatment.

## Supplementary Material

Fig_S1_(2)_ioac125

Fig_S2_ioac125

TableS1_ioac125

TableS2_ioac125

TableS3_ioac125

## Data Availability

The data underlying this article will be shared on reasonable request to the corresponding author.

## Abbreviations

PCOS, polycystic ovary syndrome; T2D, type 2 diabetes; CVD, cardiovascular disease; WAT, white adipose tissue; BAT, brown adipose tissue; UCP1, uncoupling protein 1; FGF21, fibroblast growth factor 21; FNDC5, fibronectin type III domain-containing protein 5; ITGAV, integrin alpha V; PGC-1 alpha, peroxisome proliferator-activated receptor-gamma coactivator 1 alpha; DHEA, dehydroepiandrosterone; T, total testosterone; OGTT, oral glucose tolerance test; ITT, insulin tolerance test; LH, luteinizing hormone; FSH, follicle stimulating hormone; AMH, anti-Müllerian hormone; hCG, human chorionic gonadotropin; SUV, standard uptake value; 18F-FDG, 18F-fluorodeoxyglucose; IR, insulin resistance; WB, western blotting
