# Supplementary figures and images for: Irisin reduces the abnormal reproductive and metabolic phenotypes of PCOS by regulating the activity of brown adipose tissue in mice
Source: Biol Reprod. 2022 Jun 17;107(4):1046–58. doi: 10.1093/biolre/ioac125 (PMC9562123; doi:10.1093/biolre/ioac125)

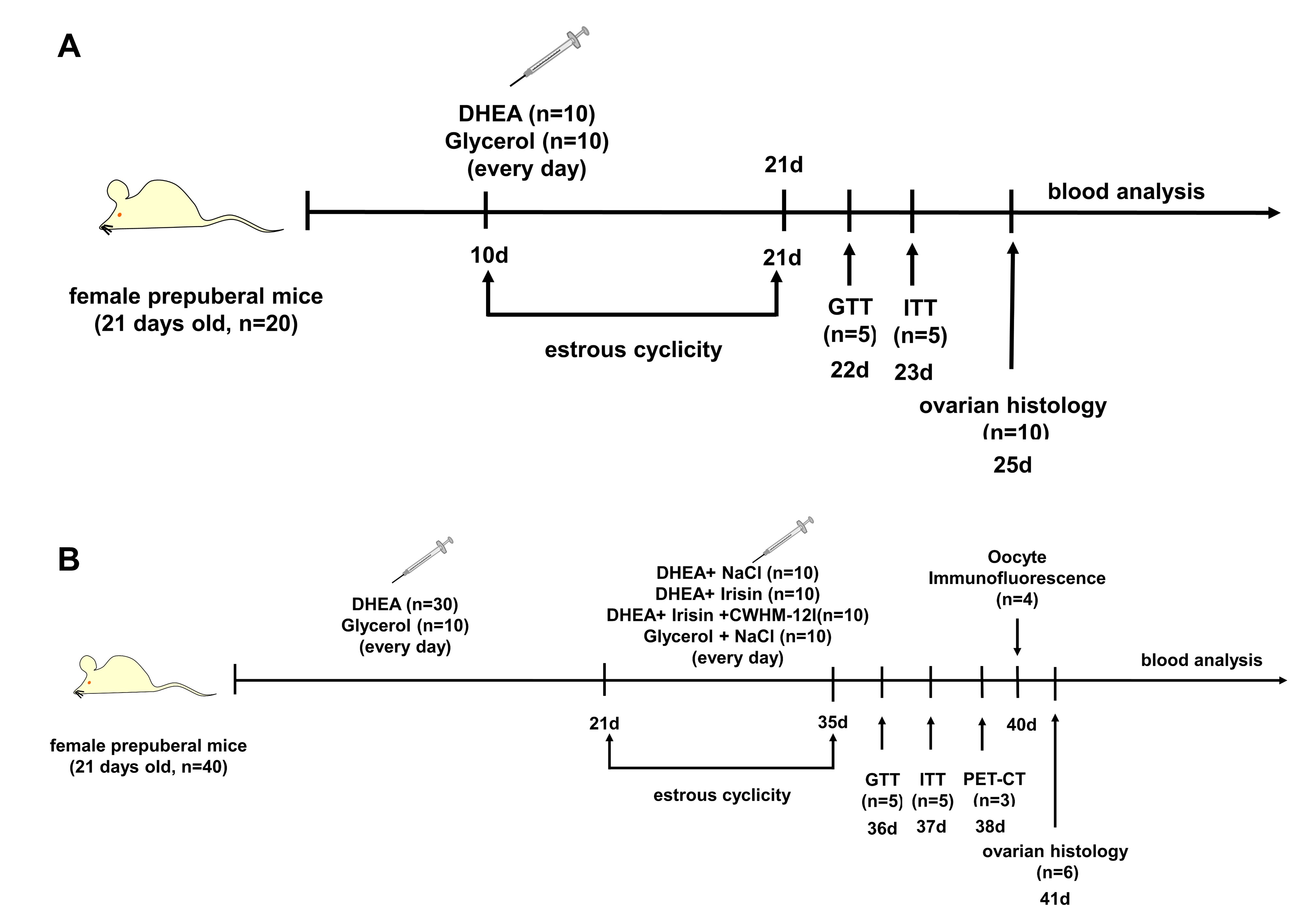

Supplement: Fig_S1_(2)_ioac125 [file fig_s1_(2)_ioac125.jpeg]

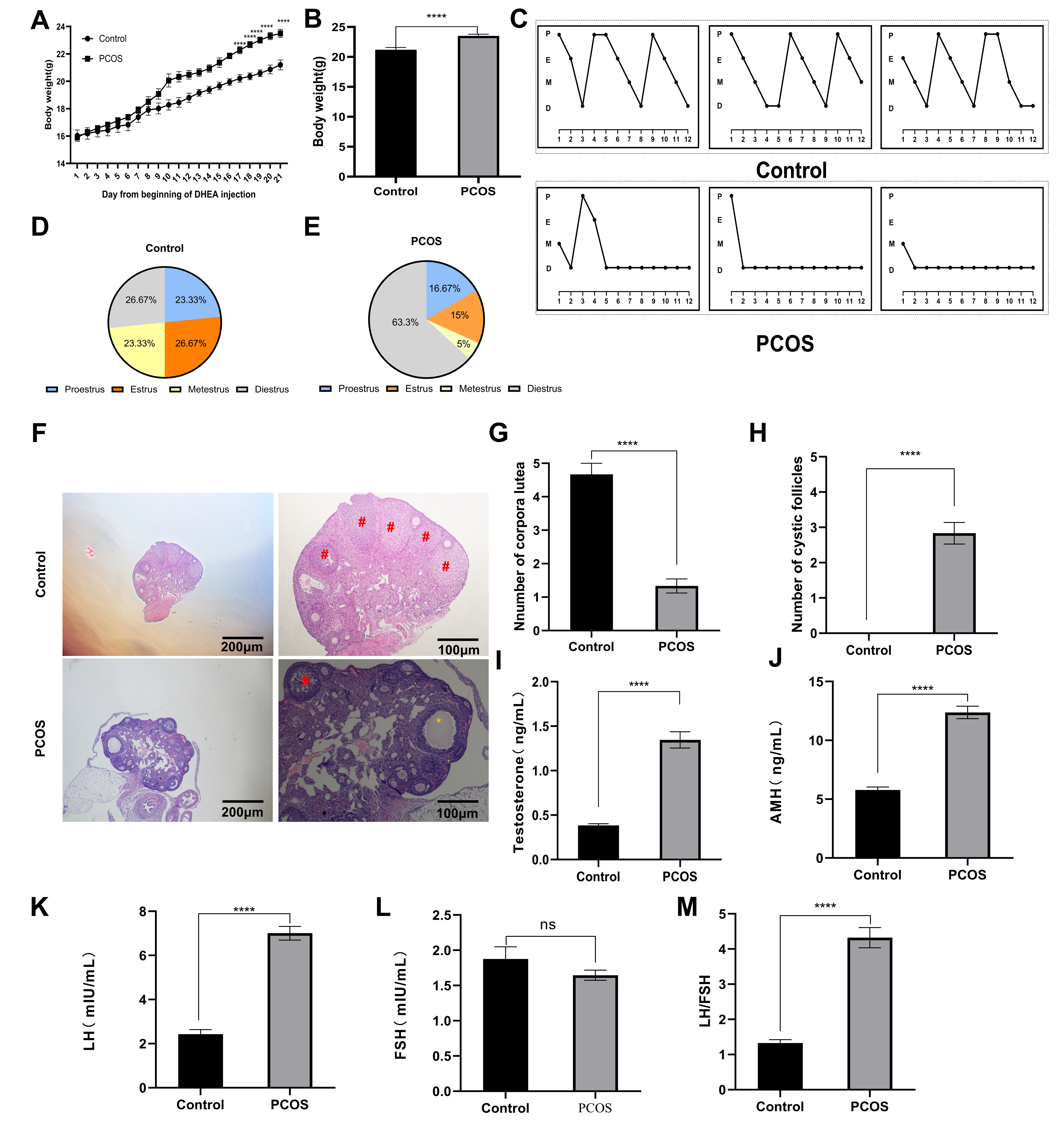

Supplement: Fig_S2_ioac125 [file fig_s2_ioac125.zip › Fig_S2_ioac125.tif]

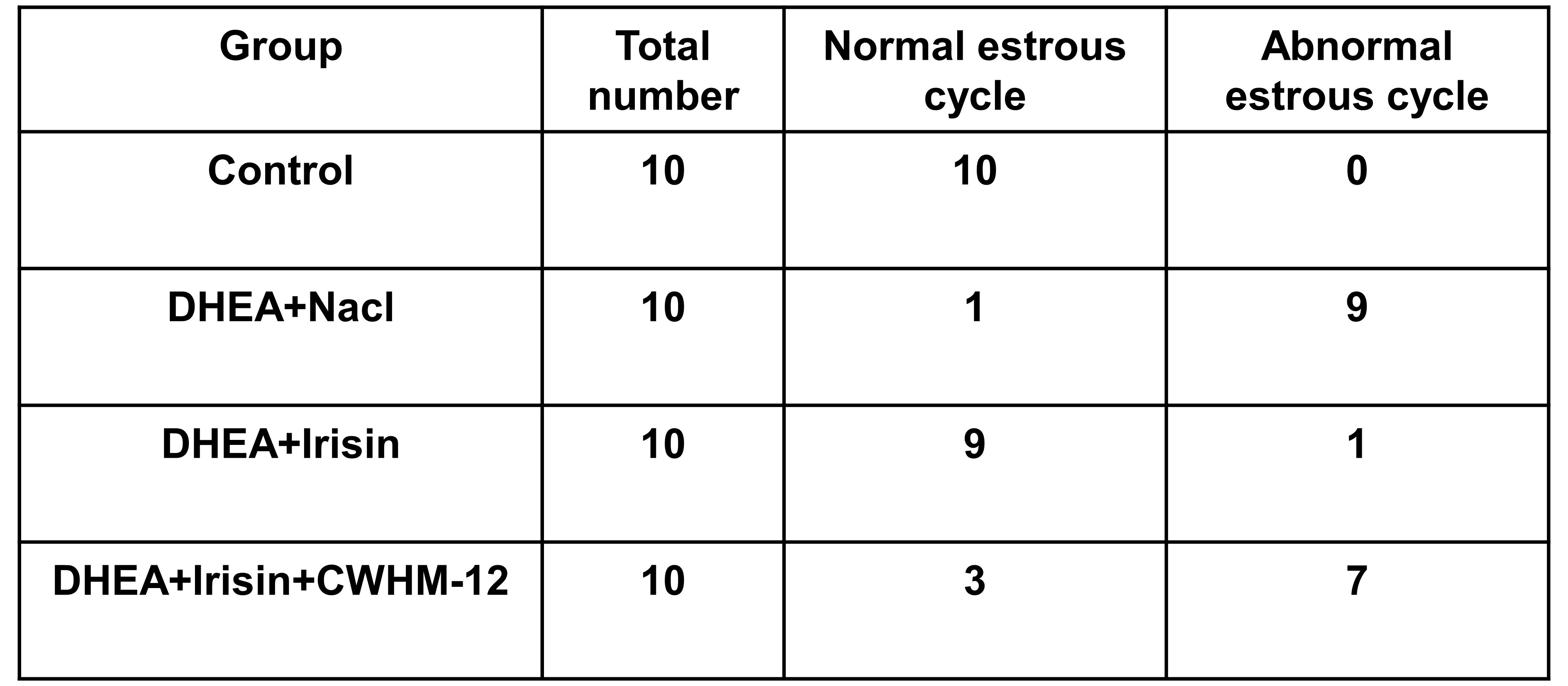

Supplement: TableS1_ioac125 [file tables1_ioac125.jpeg]

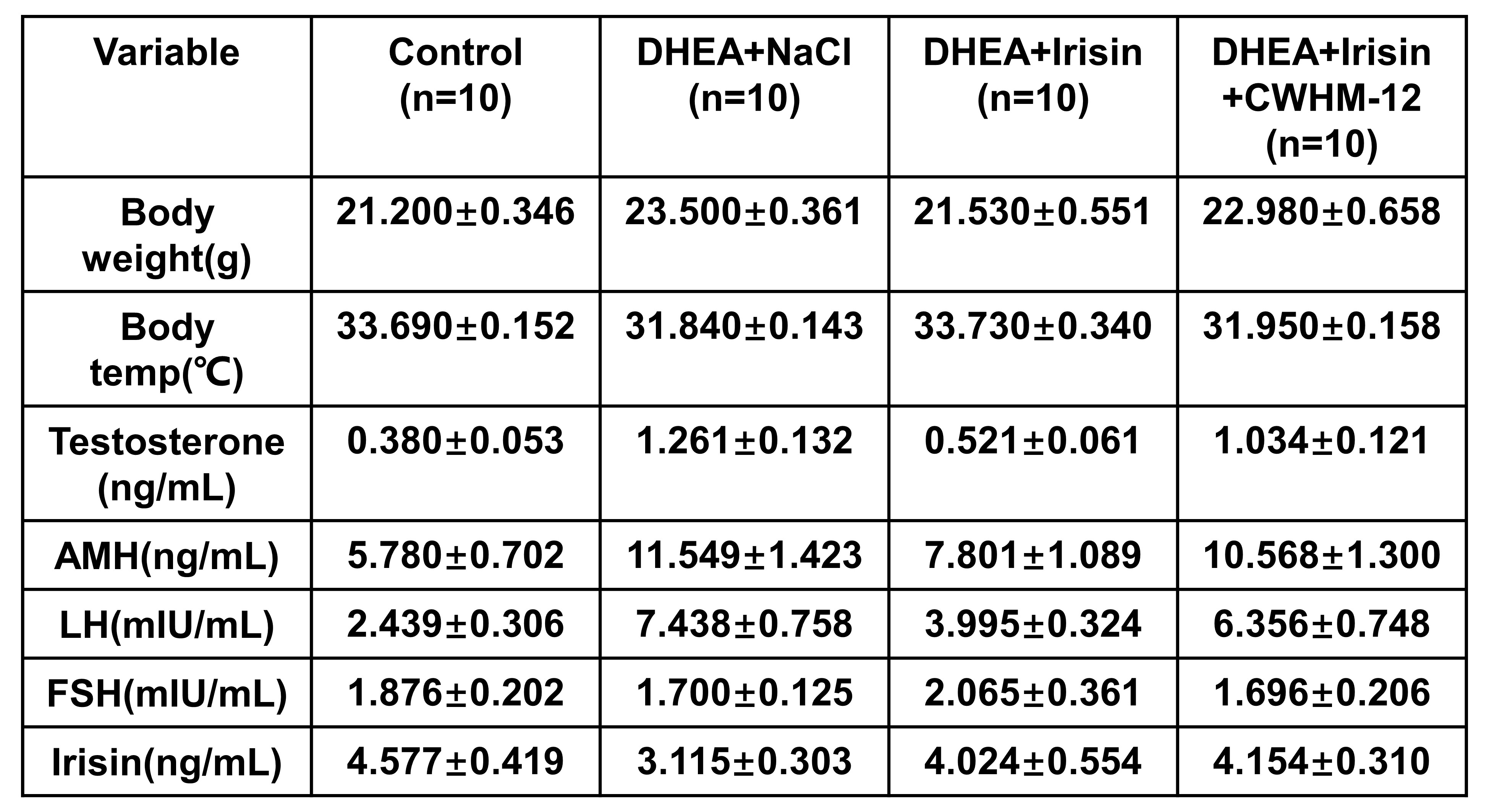

Supplement: TableS2_ioac125 [file tables2_ioac125.jpeg]

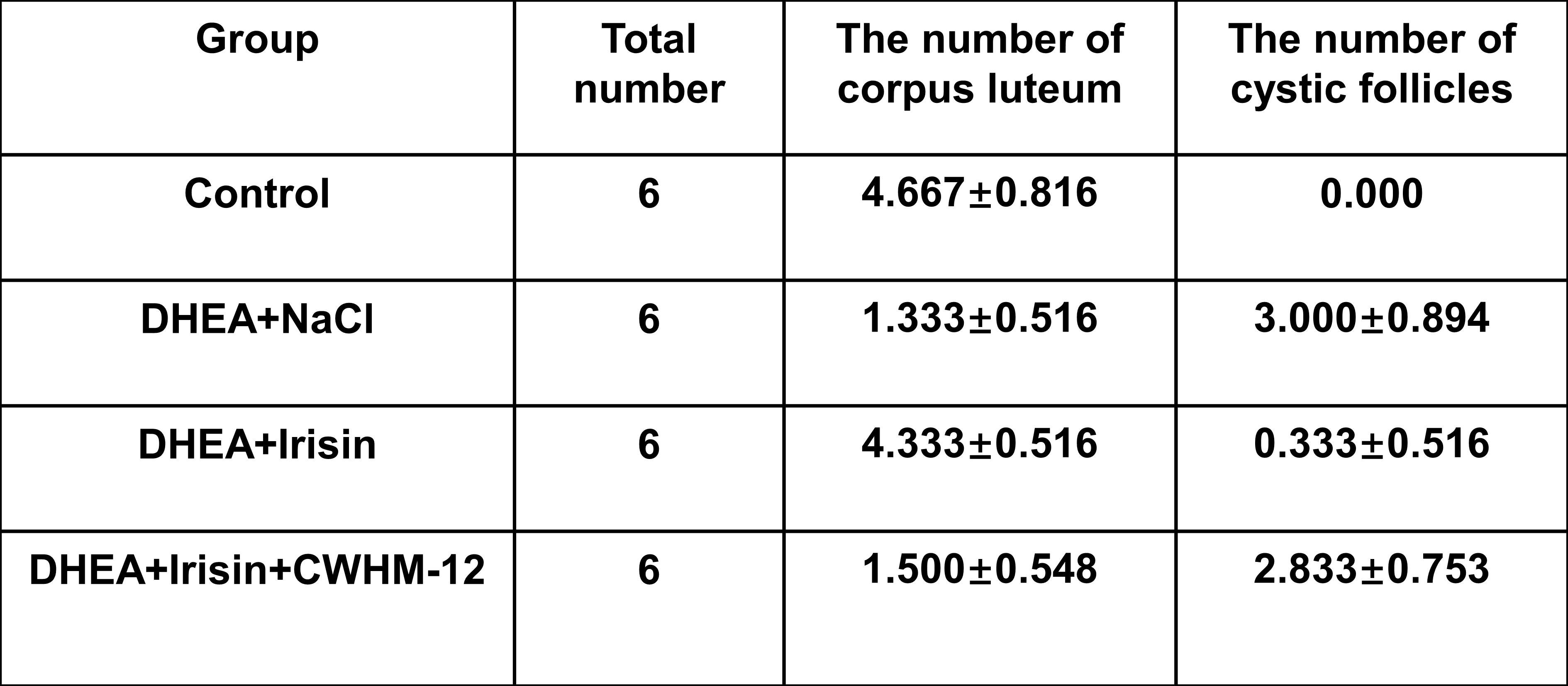

Supplement: TableS3_ioac125 [file tables3_ioac125.jpeg]
